# Supplementary material for: Enhanced voltage generation through electrolyte flow on liquid-filled surfaces
Source: Nat Commun. 2018 Oct 3;9:4050. doi: 10.1038/s41467-018-06297-9 (PMC6170469; doi:10.1038/s41467-018-06297-9)
Supplement: Supplementary file 1 — Supplementary Information [file 41467_2018_6297_MOESM1_ESM.pdf]

Supplementary Information

Enhanced voltage generation through electrolyte flow on liquid filled surfaces

Fan *et al.*

Contents

Supplementary Note 1. Geometrical parameters for the construction of air filled surfaces (*AFS)* and liquid filled surfaces (*LFS*)

Supplementary Note 2. The pressure dependence of V_s and the numerical estimation of the surface potential (ζ)

Supplementary Note 3. Estimates of the overall slip length (b_{eff}) from averaged surface potential (ζ)

Supplementary Note 4. Pressure-driven electrokinetic flow with *no* hydrodynamic slip

Supplementary Note 5. Hydrodynamic flow with no surface charge but slip at the oil-electrolyte interface

Supplementary Note 6. Considerations related to the figure of merit (in mV/Pa) and energy conversion efficiency

Supplementary Note 1: Geometrical parameters for the construction of air filled surfaces (*AFS)* and liquid filled surfaces (*LFS*):

For a *LFS* with a given $\phi_{solid} = 0.5$, data from more detailed measurements indicates that a groove width of ~ 18 μm is close to optimal for obtaining the largest V_s see Supplementary Figure 1(a), while the V_s was larger for the reported groove/trench height of 95 μm: see Supplementary Figure 1(b). It was noted that heights > 95 μm lead to fragile structures.

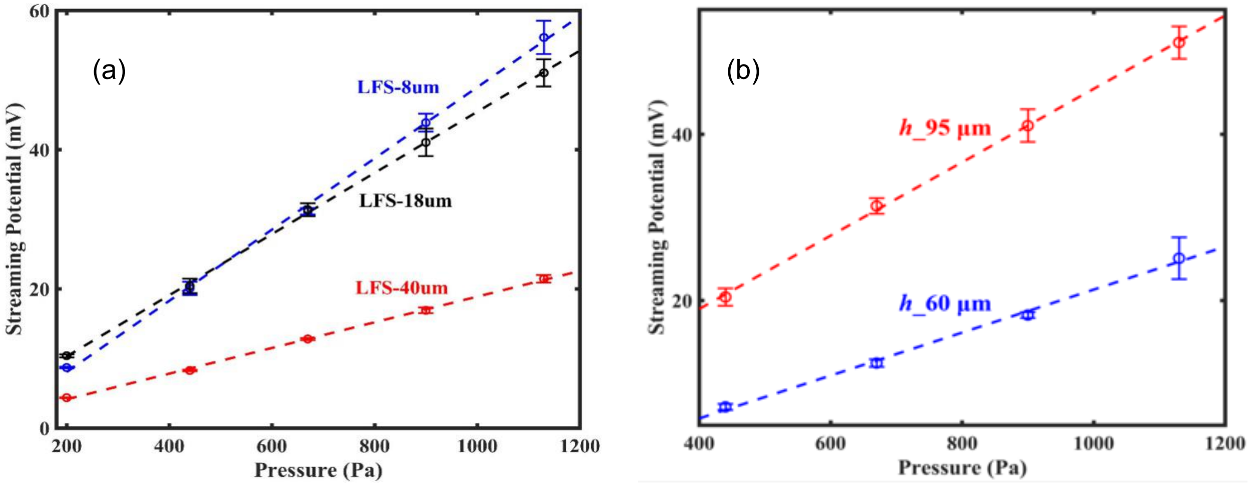

Supplementary Figure 1 The obtained streaming potential (V_s) as a function of the **(a)** groove width (w) - for 8 μm, 18 μm, and 40 μm, and **(b)** groove/trench height (h) - for 60 μm and 95 μm. In our paper, we report the results using $w = 18$ μm, and a $h = 95$ μm.

We have also noted that for a given ΔP and ϕ, that the V_s increases with the number of periods of grooves. Further work is needed to critically understand such results, as well as the precise geometrical conditions related to the optimization, and we hope that our experimental work will simulate further inquiry, from the community, into considering such aspects.

For the influence of surface geometry alone, we compare the results of *Flat* (unpatterned surface), and the air-filled surfaces (*AFS*): $Air_{0.5}$ and $Air_{0.75}$, as indicated in Figures 2(c) and (d). The air in the *AFS* can reduce the friction between the flowing liquid electrolyte and patterned surface which helps in the increase of the streaming potential (V_s). However, a non-charged liquid-air interface will have no contribution to streaming potential[1,2]. Consequently, the V_s of $Air_{0.5}$ is only a little larger than compared to the *Flat* case and the V_s of $Air_{0.75}$ is even smaller, presumably

due to an increase of non-charged liquid-air areas.

The aspect of nano- and micro-scale roughness was considered and found to be of much less importance compared to the groove width. The nominal roughness of the parylene coated surface was found to be R_a = 1.25 nm ± 0.19 nm (as determined through Dektak 150 Surface Profilometer) and the obtained streaming potential was insensitive to variations around such values. Generally, a roughness of < 6 nm has minimal effects on the slip length and flow rate[3], and can be considered hydrodynamically smooth[4].

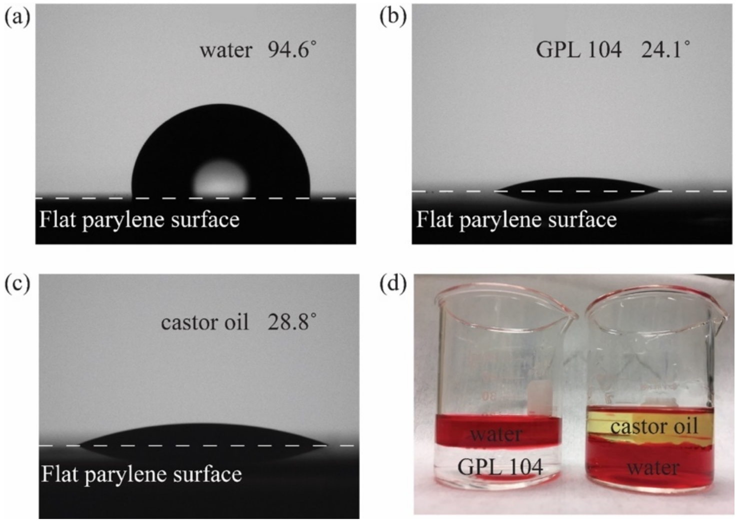

Supplementary Figure 2 The contact angle of (a) water, (b) GPL 104, and (c) castor oil on parylene coated flat Silicon surface were measured using Ramé-Hart Model 190 Contact Angle Goniometer. The measured results are 94.6°, 24.1°, 28.8° for water, GPL 104 and castor oil, respectively. It is then clear that parylene is hydrophobic (/oleophilic). (d) shows that the GPL 104 oil as well as the castor oil is immiscible with water.

Supplementary Table 1 | Physical properties of filled fluids (*data from* DuPont® Performance Lubricants, 2015)

**Oils**	**Density (g/cm^3)**	**Kinematic Viscosity (cm^2/s)**	**Dynamic Viscosity (mPa·s)**	**Dielectric Constant**	**Surface Tension (mN/m)**
Krytox® 104	1.93	1.77	340	2.1	18
Molivera Organics Castor Oil	0.96	3.25	312	4.7	39

Supplementary Note 2. The pressure dependence of V_s and the numerical estimation of the surface potential (ζ)

In the chosen pressure range, that the V_s was linearly proportional to the applied ΔP, following the Helmholtz-Smoluchowski relation: $V_S = \frac{\epsilon\zeta}{\eta\kappa}\Delta P$, as shown in Figures 2 (c), 3 (b), and 3 (c). From the slope of the V_s - ΔP curve, we estimate the surface potential (ζ) at the edge of the shear plane, assuming an the electrolyte permittivity: ε (= $\varepsilon_o\varepsilon_r$) with ε_r as the relative permittivity, *e.g.,* ~ 80 for 0.1 mM NaCl solution), η: the dynamic viscosity of the electrolyte (~ 10^{-3} Pa·s), and a bulk electrolyte conductivity (κ ~ 10^{-3} S/m), which are listed in Supplementary Table 2, below.

Supplementary Table 2 | Linear fitting results and ζ

Sample	R^2	Slope (mV/Pa)	ζ (mV)
Flat	0.9972	0.03081	44.2
$Air_{0.5}$	0.9997	0.03117	46.1
$Air_{0.75}$	0.9963	0.0286	41.0
$GPL_{0.5}$	0.9996	0.04392	65.6
$Castor_{0.5}$	0.9963	0.02418	34.7

$Air_{0.5}$: patterned air filled surface (*AFS*) with solid fraction (ϕ_s) of 0.5 and air fraction (ϕ_{air}) of 0.5

$Air_{0.75}$: *AFS* with ϕ_s of 0.25 and ϕ_{air} of 0.75

$GPL_{0.5}$: liquid filled surface (*LFS*) with Krytox GPL 104 oil, ϕ_s of 0.5 and ϕ_{GPL} of 0.5

$Castor_{0.5}$: *LFS* with castor oil, ϕ_s of 0.5 and ϕ_{Castor} of 0.5

Supplementary Note 3. Estimates of the overall slip length (b_{eff}) from averaged surface potential (ζ)

The ζ values listed in Supplementary Table 2, may be considered an averaged/effective value($= \zeta_{eff}$) considering the top (*PDMS*) and bottom (the *LFS*, constituted from the *parylene* and the *oil* in the *interstices*) surfaces of the channel. Based on the effective medium approach (EMA)[1], we consider a weighted summation of the ζ_{PDMS}, $\zeta_{parylene}$, and $\zeta_{interstice}$ as follows:

$$\zeta_{eff} = 0.5\zeta_{PDMS} + 0.5\zeta_{parylene} \qquad \text{(S3-1)}$$

$$\zeta_{eff} = 0.5\zeta_{PDMS} + \frac{b_{eff}}{\lambda_D}(0.25\zeta_{parylene} + 0.25\zeta_{interstice}) \qquad \text{(S3-2)}$$

The reported $\zeta_{parylene}$ is 17.5 mV at 10 mM NaCl solution[5], consequently $\zeta_{parylene}$ was estimated to be ~ 35 mV for the 0.1 mM electrolyte used in our studies, based on the relation[6] $\zeta \sim \log[I]$. The ζ_{PDMS} was estimated to be ~ 53.4 mV based on the first equation using $\zeta_{eff} = 44.2$ mV for *Flat*. Such an estimate for the ζ_{PDMS} seems to be in reasonable accord with literature, considering that there may be many affecting factors[6,7], *e.g.,* counter-ion type, pH of the solution and temperature. The $\zeta_{interstice} = 0$ mV for air[1]. Then, the b_{eff} for $Air_{0.5}$ can be estimated as 66.5 nm based on the second equation. Given that[8] the ζ_{oil} is ~ 69 mV, the effective slip length b_{eff} for the $GPL_{0.5}$: would be ~ 44.9 nm. The b_{eff} can also be estimated for $Air_{0.25}$ and $Castor_{0.5}$ using the same method and all the results are listed in Supplementary Table 3.

Supplementary Table 3 | Estimates of the *effective* slip length: b_{eff}

**Sample**	b_{eff}**(nm)**
$Air_{0.5}$	66.5
$Air_{0.25}$	98.0
$GPL_{0.5}$	44.9
$Castor_{0.5}$	9.2

The b_{eff} of $Air_{0.75}$ is larger compared to that of $Air_{0.5}$ and maybe related to the larger amount of air, in the former. The b_{eff} of *GPL oil* is smaller due to a larger viscosity, compared to air, while the b_{eff} of *castor oil* is even smaller due to a larger dielectric constant.

While significant care was taken in removing the oil from the surface in the use of the *LFS*, there is a possibility, as pointed out by the reviewer, that the oil may partially cover the parylene surface. Consequently, the effective medium approach (EMA) aspect may indeed be considered an approximation. Previously, we used $\phi_{parylene}$ = 0.25 and $\phi_{interstice}$ = 0.25 (if parylene area was not covered by oil) and obtained an effective slip length (b_{eff}) of ~ 44.9 nm for $GPL_{0.5}$. However, even with $\phi_{parylene}$ = 0.1 and $\phi_{interstice}$ = 0.4 (if 80% of the LFS was covered by oil), the b_{eff} is ~ 37.5 nm, a change of ~ 16%. Consequently, the use of the EMA seems to be a reasonable approximation.

Supplementary Note 4. Pressure-driven electrokinetic flow with *no* hydrodynamic slip

To describe electrolyte flow, we modeled through self-consistent finite element simulations (COMSOL®), the local fluid velocity at a point ($\boldsymbol{u}$), the respective ionic species concentration (c_i), and electric potential (ϕ). The relevant mass conservation equations, and the Stokes equation were coupled with the Nernst-Planck-Poisson (*N-P-P*) models, as described below.

Let i= 1, 2 represent the ionic species present in the electrolyte, *e.g., i*=1 refers to Na^+, while i=2, refers to the Cl^- species. The flux conservation equation[9] of each ionic species, i, is

$$\frac{\partial n_i}{\partial t} = \nabla \cdot \boldsymbol{J_i} \tag{S4-1}$$

where $\boldsymbol{J_i}$ is the ionic flux vector and n_i is the number density of ions per unit volume. $n_i = c_i * N_{Av}$ where c_i is the concentration of each ionic species in moles/L (M) and N_{Av} is the Avogadro constant = 6.02 x10^{23} mole^{-1}. For steady state flow: $\nabla \cdot \boldsymbol{J_i} = 0$.

The Nernst-Planck (*N-P*) equation[10–12]: Eqn. (S4-2) below, considers the motion of ions in a fluid as a function of fluxes due to convection, diffusion, as well as ion migration under the effects of electrostatic forces.

$$\boldsymbol{J_i} = n_i\boldsymbol{u} - D_i\nabla n_i - \frac{z_i e\, n_i D_i}{k_B T}\nabla\phi \tag{S4-2}$$

D_i is the respective species diffusivity, *i.e.,* $D_{Na^+} = 1.13 \times 10^{-9}$ m^2 /s and $D_{Cl^-} = 1.0 \times 10^{-9}$ m^2 /s. z_i is the valence of each ionic species. $z_{Na^+} = 1$ and $z_{Cl^-} = -1$, k_B is the Boltzmann constant = 1.38 x 10^{-23} J/K and T = 298 K. The $\boldsymbol{u}$ is obtained through the Stokes' equation[13]: Eqn. (S4-3),

$$-\nabla p + \mu\nabla^2\boldsymbol{u} + \rho_f\nabla\phi = 0 \tag{S4-3}$$

Here, pressure gradient driving the flow (∇p), the viscous force ($\mu\nabla^2 u$, with μ as the dynamic viscosity) and the electrical body force (=$\rho_f\nabla\phi$), with ρ_f as the volumetric space charge density in the electrolyte due to the presence of free charges, *e.g.,* counter-ions near the surface, are all considered. The electric potential (ϕ) is obtained from the Poisson (*P*) equation[14]:

$$\nabla \cdot (\epsilon_0\epsilon_r\nabla\phi) = \rho_f \tag{S4-4}$$

ϵ_0 is = 8.854·10^{-12} C^2/Nm^2- the permittivity of free space and $\epsilon_r$is the relative permittivity of the species near the wall. The ϕ from Eqn. (S4-4) and the n_i from Eqn. (S4-2) are coupled through:

$$\rho_f = \sum_{i=1}^{2} z_i e\, n_i \quad \text{(S4-5)}$$

Eqns. (S4-2), (S4-3), (S4-4), and (S4-5) are solved self-consistently, and used to obtain (i) $\boldsymbol{u}$, and (ii) ϕ and related n_i, at any point (x, y) in the channel. Here, x is along the direction of fluid flow along the length of the channel, and y is the vertical coordinate – along the height of the channel. The $\boldsymbol{u}$ yields the local velocity and is averaged along the width of the channel for the average velocity. The $V_{s,j}$ developed between any two points (x_j, y_j) and (x_{j+1}, y_j) along the channel is then computed from $|\phi\ (x_j,\ y_j)\ -\ \phi\ (x_{j+1},\ y_j)|$. The net V_s was aggregated from such individual contributions along the length of the channel. The mesh size, for the finite element simulations, was chosen to be 5 nm at both the top and bottom walls outwards.

Boundary Conditions: For the N-P equation (Eqn. S4-2), the bulk concentration $\boldsymbol{c_o} (= \boldsymbol{c_{Na^+}} = \boldsymbol{c_{Cl^-}})$ = 0.1 mM, was kept constant for both the electrolyte ion species at both inlet and outlet, as well as at the centerline of the channel, assumed to be far outside the electrical double layer (EDL), as the Debye length ($\boldsymbol{\lambda_D} = \sqrt{\frac{\boldsymbol{\epsilon_o \epsilon_r k_B T}}{\boldsymbol{e^2 c_i}}}$) was estimated to be ~ 30 nm. The difference of ionic concentration between counter-ions and co-ions, yields a net charge profile density along the height of the channel. The flux normal to top and bottom walls $\boldsymbol{J_i} = \boldsymbol{0}$.

For the Poisson equation (Eqn. S4-4), the surface charge density (σ) at the top wall[15]: σ_{PDMS} = -18 mC/m^2, while at the bottom surface, (i) at the oil[8]-electrolyte interface is σ_{oil}= -1.8 mC/m^2, and (ii) for the parylene[16]-electrolyte interface is $\sigma_{Parylene}$ = - 3.7 mC/m^2. The σ values are related to the surface electrical field. The outlet was set at zero potential (/ground). It was assumed that the electric field due to EDL and that due to generated streaming potential were completely decoupled. The ϕ at the center-line of flow, in the middle of the channel, *i.e.,* $\phi\ (y = H/2) = 0$ and far away from the EDL at the surface was set to zero.

For the Stokes equation (Eqn. S4-3), the inlet pressure was set to 1200 Pa, while the outlet was set to zero. The no-slip hydrodynamic boundary condition was assumed to hold true at all interfaces.

Supplementary Note 5. Hydrodynamic flow with no surface charge but slip at the oil-electrolyte interface

Here, the Stokes Equation (without any electrical body force) is the governing equation

$$-\nabla p + \mu \nabla^2 u = 0 \qquad \text{(S5-1)}$$

The inlet pressure was set to 1200 Pa, while the outlet was set to zero. A Navier slip boundary condition: $u_s\,(y=0) = b\,\frac{\partial u\,(y=0)}{\partial y}$ over the flowing electrolyte-oil interface was assumed, with a finite slip velocity: u_s and slip length: b). For a hydrophobic surface the b may be approximated[17,18] to be at least d, *i.e.*, ~ 18 μm.

Supplementary Note 6. Considerations related to the figure of merit (in mV/Pa) and energy conversion efficiency

Our focus, in the presented work, was less on the energy conversion efficiency and *more* on methodologies to enhance the streaming potential (V_s) per unit pressure difference (ΔP), *i.e.,* $\left(\frac{V_S}{\Delta P}\right)$. Indeed, our work has experimentally demonstrated the *largest figure of merit*, thus far, in terms of the voltage generated per unit applied pressure, in comparison to previous experimental work[19–22].

More specifically, the fluid flow to electrical conversion efficiency (*Eff.*) = P_{out} /P_{in}, where the output power: P_{out} (= $\frac{1}{4}V_S \cdot I_S$), I_S (= $V_S\frac{A\sigma}{L}$) is the streaming current, with A (= $w \cdot h$) as the cross-sectional area of the channel of width: w, and height: h, and $P_{in} = Q \cdot \Delta P$, Q is the flow rate (= $\frac{Gh^3}{12\eta}$), with G as a constant pressure gradient, and η the viscosity – general formulation adapted from Olthuis, *et al, Sensors and Actuators B*, vol. 111-112, p. 385, (2005).

Then, the $Eff. = \frac{3V_S^2\sigma\eta w}{\Delta PLGh^3} = 3\left(\frac{V_S}{\Delta P}\right)\left(\frac{V_S}{L}\right)\left(\frac{\eta\sigma}{G}\right)\left(\frac{w}{h^2}\right)$. For a given electrolyte concentration (fixed σ) and flow velocity (a given u), *both* $\left(\frac{V_S}{\Delta P}\right)$ and $\left(\frac{w}{h^2}\right)$are important. While the former, *i.e.,* $\left(\frac{V_S}{\Delta P}\right)$ was considered in detail in our work, the geometrical factor: $\left(\frac{w}{h^2}\right)$ may be significantly enhanced through the use of nanoscale diameter channels (small h) with the overlap of double layers, as seen for example, in the work by van der Heyden, *et al, Nanoletters,* vol. 6, p. 2232, (2006). We used channels of the order of 250 μm in height over which most of the electric field is zero and a finite field is obtained only close to the channel surfaces of the order of 0.1 μm (the Debye length). Consequently, the estimated energy conversion efficiencies in our work is quite small of the order 10^{-3} %, employing the computational methodology indicated in van der Heyden, *et al, Nanoletters,* vol. 6, p. 2232, (2006). More specifically, the efficiency values are ~ $7.7 \cdot 10^{-4}$ % (through the use of the oil in a *LFS*), ~ $3.8 \cdot 10^{-4}$ % (on an *AFS*), and ~ $3.5 \cdot 10^{-4}$ % (on a flat unpatterned substrate).

An important point to note is that the energy conversion efficiency may be improved by more than a factor of two through the use of LFS compared to the flat/AFS substrates. We may expect that

the efficiency of nanochannels may be further enhanced by using *LFS*, and will be one of the focus areas of future work.

Supplementary References

1. Squires, T. M. Electrokinetic flows over inhomogeneously slipping surfaces. *Phys. Fluids* **20,** 092105 (2008).
2. Bahga, S. S., Vinogradova, O. I. & Bazant, M. Z. Anisotropic electro-osmotic flow over super-hydrophobic surfaces. *J. Fluid Mech.* **644,** 245–255 (2010).
3. Zhu, Y. & Granick, S. Limits of the Hydrodynamic No-Slip Boundary Condition. *Phys. Rev. Lett.* **88,** 106102 (2002).
4. Cottin-Bizonne, C., Cross, B., Steinbeger, A. & Charlaix, E. Boundary Slip on Smooth Hydrophobic Surfaces: Intrinsic Effects and Possible Artifacts. *Phys. Rev. Lett.* **94,** 056102 (2005).
5. Goda, T., Konno, T., Takai, M. & Ishihara, K. Photoinduced phospholipid polymer grafting on Parylene film: Advanced lubrication and antibiofouling properties. *Colloids Surfaces B Biointerfaces* **54,** 67–73 (2007).
6. Kirby, B. J. & Hasselbrink, E. F. Zeta potential of microfluidic substrates: 1. Theory, experimental techniques, and effects on separations. *Electrophoresis* **25,** 187–202 (2004).
7. Beattie, J. K. The intrinsic charge on hydrophobic microfluidic substrates. *Lab Chip* **6,** 1409 (2006).
8. Marinova, K. G. *et al*. Charging of Oil−Water Interfaces Due to Spontaneous Adsorption of Hydroxyl Ions. *Langmuir* **12,** 2045–2051 (1996).
9. Probstein, R. F. *Physicochemical Hydrodynamics: An Introduction*. (John Wiley & Sons Inc., 1994).
10. Hunter, R. J. *Zeta potential in colloid science: Principles and Applications*. (Academic Press, 1986).
11. Dukhin, S. S. Development of Notions as to the mechanism of electrokinetic phenomena and teh structure of the colloid micelle. in *Surface and Colloid Science* 1–47 (John Wiley & Sons, 1974).
12. Bard, A. J. & Faulkner, L. R. *Electrochemical Methods: Fundamentals and Applications, 2nd Ed. John Wiley and Sons* (2001).
13. Kundu, P. K. & Cohen, I. M. *Fluid Mechanics. Review Literature And Arts Of The Americas* (2008). doi:978-0-12-381399-2
14. Jackson, J. D. *Classical Electrodynamics*. (John Wiley, 1999).

15. Schrott, W. *et al*. Study on surface properties of PDMS microfluidic chips treated with albumin. *Biomicrofluidics* **3,** (2009).

16. Lo, H. W. & Tai, Y. C. Parylene-based electret power generators. *J. Micromechanics Microengineering* **18,** (2008).

17. Rothstein, J. P. Slip on Superhydrophobic Surfaces. *Annu. Rev. Fluid Mech.* **42,** 89–109 (2010).

18. Ybert, C., Barentin, C., Cottin-Bizonne, C., Joseph, P. & Bocquet, L. Achieving large slip with superhydrophobic surfaces: Scaling laws for generic geometries. *Phys. Fluids* **19,** (2007).

19. van der Heyden, F. H. J., Bonthuis, D. J., Stein, D., Meyer, C. & Dekker, C. Power Generation by Pressure-Driven Transport of Ions in Nanofluidic Channels. *Nanoletters* **7,** 1022–1025 (2007).

20. Xie, Y., Sherwood, J. D., Shui, L., van den Berg, A. & Eijkel, J. C. T. Strong enhancement of streaming current power by application of two phase flow. *Lab Chip* **11,** 4005–4011 (2011).

21. Xie, Y. *et al*. Electric energy generation in single track-etched nanopores. *Appl. Phys. Lett.* **93,** 163116 (2008).

22. Li, Z.-Y., Liu, Y., Zheng, Y.-Y. & Xu, R.-K. Zeta potential at the root surfaces of rice characterized by streaming potential measurements. *Plant Soil* **386,** 237–250 (2015).
